# The Effect Of Pet Therapy On Anxiety In Anxiety Disorders: A Systematic Review

**DOI:** 10.1192/j.eurpsy.2025.1035

**Published:** 2025-08-26

**Authors:** M. T. Sinan, C. H. Ayhan, M. C. Aktaş, H. Akbulut

**Affiliations:** 1 Mental Health And Pschiatric Nursig, Van Yuzuncu Yil University, VAN, Türkiye

## Abstract

**Introduction:**

Observation of the potential benefits of human-animal interaction is evident. Pet therapy is used to treat anxiety as it significantly reduces self-reported anxiety in many age groups. Most studies of pet therapy involve dogs, but cats and horses have also been studied. Pet therapy is becoming increasingly popular and is used in a variety of diverse ways, from promoting communication in older adults to improving the wellbeing of those with serious mental illness. Studies have also found mental health inpatients with mood and psychotic disorders to display significant reductions in anxiety on the State-Trait Anxiety Inventory.

**Objectives:**

This study aims to measure the effect of pet therapy (especially therapy with pets such as dogs or cats) in individuals with anxiety disorders. It is aimed to examine how pet therapy affects anxiety levels, whether it reduces anxiety symptoms and its contribution to overall quality of life.

**Methods:**

The study will be conducted between November 2024 and January 2025 in 3 databases (PubMed, Cochrane Library, Science Direct) using the keywords “anxiety disorders”, “anxiety”, “pet therapy”. These databases were preferred because they contain a significant amount of evidence-based literature in biomedical sciences and psychology. Studies conducted between 2015 and 2024, whose full texts were accessed and written in Turkish and English were included in the study.

**Results:**

There are 20 national and international research articles on the subject and the literature review is ongoing. When the literature review is completed, all study results will be presented together.

**Conclusions:**

This study provides an overview of the positive effects of pet therapy in reducing anxiety symptoms and improving quality of life in individuals with anxiety disorders. In the studies reviewed, it was found that therapy with pets, especially dogs and cats, led to significant reductions in anxiety levels and improved individuals’ overall mental health. Many of the participants felt calmer, less stressed and had higher levels of satisfaction with the therapy. The findings suggest that pet therapy not only reduces anxiety levels, but also increases social communication, reduces feelings of loneliness and improves functioning in daily life. Pet therapies can be considered as a complementary method for anxiety reduction.

**Disclosure of Interest:**

None Declared

